# *Ab Initio* Path Integral Monte Carlo Simulations of the Uniform Electron Gas on Large Length Scales

**DOI:** 10.1021/acs.jpclett.3c03193

**Published:** 2024-01-29

**Authors:** Tobias Dornheim, Sebastian Schwalbe, Zhandos A. Moldabekov, Jan Vorberger, Panagiotis Tolias

**Affiliations:** † Center for Advanced Systems Understanding (CASUS), Helmholtz-Zentrum Dresden-Rossendorf (HZDR), D-02826 Görlitz, Germany; ‡ Institute of Radiation Physics, Helmholtz-Zentrum Dresden-Rossendorf (HZDR), D-01328 Dresden, Germany; ¶ Space and Plasma Physics, Royal Institute of Technology (KTH), Stockholm SE-100 44, Sweden

## Abstract

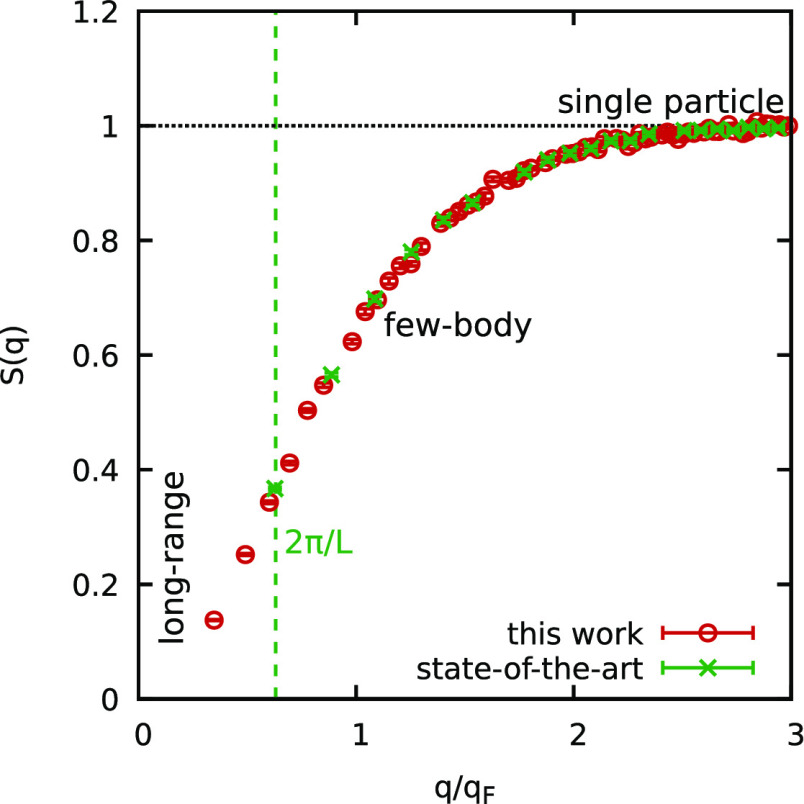

The accurate description of non-ideal quantum many-body systems is of prime importance for a host of applications within physics, quantum chemistry, materials science, and related disciplines. At finite temperatures, the gold standard is given by *ab initio* path integral Monte Carlo (PIMC) simulations, which do not require any empirical input but exhibit an exponential increase in the required computation time for Fermionic systems with an increase in system size *N*. Very recently, computing Fermionic properties without this bottleneck based on PIMC simulations of fictitious identical particles has been suggested. In our work, we use this technique to perform very large (*N* ≤ 1000) PIMC simulations of the warm dense electron gas and demonstrate that it is capable of providing a highly accurate description of the investigated properties, i.e., the static structure factor, the static density response function, and the local field correction, over the entire range of length scales.

The accurate description of Fermionic quantum many-body systems is a paramount task within physics, quantum chemistry, materials science, and related fields. An important subcategory is given by thermal simulations that describe quantum systems at finite temperatures. For example, warm dense matter (WDM),^1^ an extreme state combining high temperatures (*T* ∼ 10^4^–10^8^ K), densities (*n* ∼ 10^22^–10^27^ cm^–3^), and pressures (*P* ∼ 1–10^4^ Gbar), is ubiquitous throughout our universe and occurs in a host of astrophysical objects such as giant planet interiors^2^ and brown dwarfs.^3,4^ In addition, the fuel capsule in inertial confinement fusion applications has to traverse the WDM regime^5^ in a controlled way to reach ignition.^6^ Consequently, WDM constitutes a highly active topic and is routinely realized in experiments at large research facilities using different techniques.^7^ Other examples for thermal quantum many-body systems of Fermions include ultracold atoms^8−12^ and electrons in quantum dots.^13−16^

From a theoretical perspective, the rigorous description of such systems constitutes a difficult challenge as it must capture the complex interplay of effects such as non-ideality, quantum degeneracy, and diffraction, as well as strong thermal excitations.^1,17^ In this context, the *ab initio* path integral Monte Carlo (PIMC) method^18^ is a promising candidate as it is, in principle, capable of delivering an exact solution to the full quantum many-body problem of interest without the need for empirical input such as the exchange–correlation functional in density functional theory^19^ or the self-energy in Green function approaches.^20,21^ Unfortunately, the PIMC simulation of Fermions is afflicted with the notorious Fermion sign problem,^22^ which leads to an exponential increase in the required computation time, for example, with an increase in system size *N*.^23,24^

While a complete solution of the sign problem remains unlikely, the pressing need for an accurate description of quantum Fermi systems has ignited a number of promising developments in the field of quantum Monte Carlo simulations over the past decade, e.g., refs (25−44). On one hand, these methods allow for a very accurate description of a given *N*-body system. In combination with appropriate finite-size corrections,^31,45,46^ this has led to the first reliable parametrizations of the uniform electron gas (UEG) covering the entire WDM parameter space,^32,40,47,48^ allowing for thermal density functional theory (DFT) calculations of real WDM systems on the level of the local density approximation^49−51^ and beyond.^52,53^ On the other hand, these tools by themselves are not capable of capturing long-range phenomena^54^ that manifest on length scales larger than the given box length *L* that, for a given density, is determined by the number of simulated particles, *N*.

This precludes, for example, the description of X-ray Thomson scattering (XRTS) experiments, a key diagnostic for WDM applications,^55−58^ in a forward scattering geometry where the system is probed at a small momentum transfer *q* and, consequently, a long wavelength λ = 2π/*q*. Moreover, electrical^59^ and thermal^60^ conductivities are necessarily defined in the optical limit of *q* → 0, which makes the simulation of large systems even more important.

Very recently, Xiong and Xiong^61^ have proposed to alleviate the sign problem by carrying out path integral molecular dynamics (PIMD) simulations of fictitious identical particles governed by the continuous parameter ξ ∈ [−1, 1], with ξ = 1, ξ = 0, and ξ = −1 corresponding to the physically relevant cases of Bose, Boltzmann, and Fermi statistics, respectively. Subsequently, Dornheim et al.^62^ have implemented the same idea into PIMC and found that it works remarkably well for weakly to moderately quantum degenerate systems, including the warm dense UEG. In a nutshell, these findings imply the intriguing possibility of PIMC simulations of large Fermi systems without the exponential bottleneck due to the sign problem.

In this work, we rigorously test this hypothesis by carrying out unprecedented large-scale PIMC simulations [for which *N* ≤ 10^3^ (see Figure 1)] of the UEG both at WDM parameters, and in the strongly coupled electron liquid regime.^63,64^ In this way, we unambiguously demonstrate the capability of the ξ extrapolation method to accurately describe the system on all length scales, including the difficult long-wavelength limit of *q* → 0.

**Figure 1:**
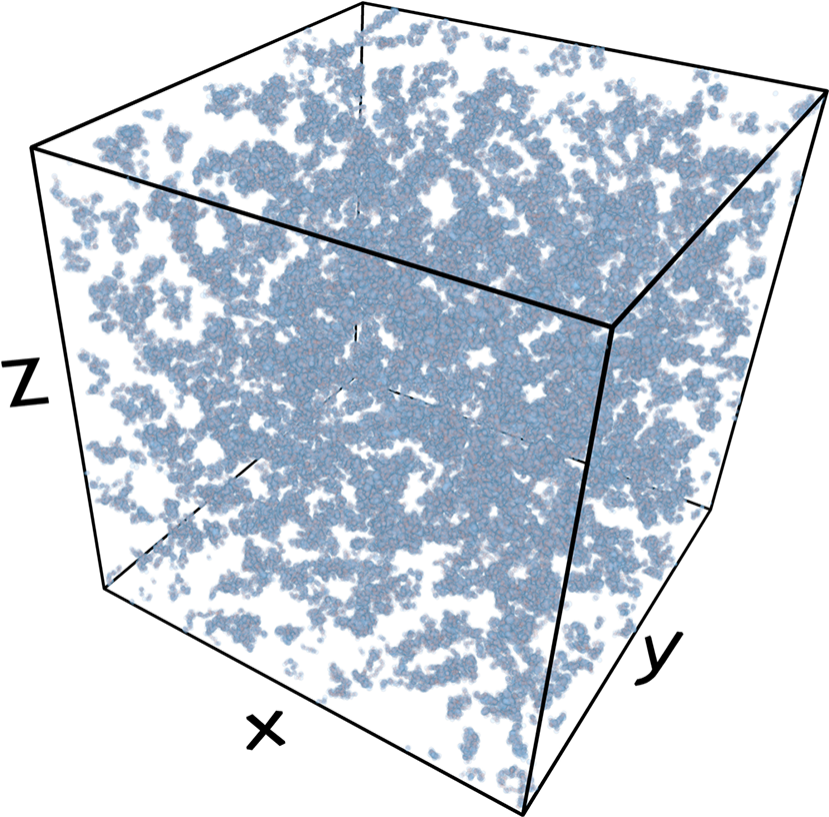
Snapshot from an *ab initio* PIMC simulation of the warm dense UEG at *r*_s_ = 2 and Θ = 1 with *N* = 1000 unpolarized electrons. Each electron is represented by an entire path along the imaginary time with *P* = 200 time steps (blue spheres); their extension corresponds to the thermal wavelength  and takes into account quantum effects such as diffraction.

These findings open up a gamut of new possibilities for studying WDM, ultracold atoms, and a host of other Fermi systems.

Let us consider a system of *N* = *N*^*↑*^ + *N*^*↓*^ unpolarized electrons in a cubic simulation cell of volume *V* = *L*^3^ at inverse temperature β = 1/*k*_B_*T*. Writing the partition function in coordinate representation then gives
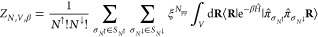
1where the meta-variable  contains the coordinates of both spin-up and spin-down electrons. Due to the indistinguishable nature of electrons of equal spin orientation, we have to sum over all possible permutations of particle coordinates that are realized by the corresponding permutation operators  with *i* ∈ {*↑*, *↓*}. A special role is played by the aforementioned continuous spin variable ξ, which effectively controls the likelihood of pair exchanges (with *N*_pp_ being the particular number of pair exchanges needed to realize a particular permutation) in the PIMC simulation.^62,65^ When ξ ≥ 0, all contributions to *Z* are positive and no sign problem occurs. We note that the special case in which ξ = 0 corresponds to the simulation of distinguishable particles, as only the term with *N*_pp_ = 0 leads to a non-zero contribution as a matter of definition (physical necessity). A more comprehensive discussion of the limit of 0° has been given in the literature.^66^ In contrast, positive and negative contributions increasingly cancel for ξ < 0, which is most severe in the Fermionic limit of ξ = −1. To avoid the associated exponential decrease in accuracy, Xiong and Xiong^61^ have proposed to extrapolate to the latter by fitting a quadratic polynomial of the form
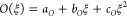
2to PIMC results for an observable *Ô* in the sign-problem free domain, i.e., ξ ≥ 0. Subsequently, Dornheim et al.^62^ have found that this approach works remarkably well for a host of observables, including energy *E*, static structure factor *S*(*q*), and even the imaginary-time density–density correlation function *F*(*q*, τ)^58,67^ if the degree of quantum degeneracy is moderate or weak. This straightforward analytical connection between bosons and Fermions breaks down in the strongly quantum degenerate limit, when quantum statistics predominantly govern the physical behavior of the system.^68^

Additional details regarding the derivation of the imaginary-time PIMC method^18^ as well as the Fermion sign problem^23,24^ have been presented in the literature and need not be repeated here. For the sake of completeness, we note that we use a canonical adaption of the worm algorithm by Boninsegni et al.^69,70^ based on the extended ensemble idea presented in ref (71). All results have been obtained for *P* = 200 imaginary-time propagators with a primitive factorization scheme, and convergence with *P* has been carefully checked.

Let us start our investigation by considering the UEG at a Wigner–Seitz radius *r*_s_ = 2 and degeneracy temperature Θ = *k*_B_*T*/*E*_F_ = 1 (where *E*_F_ is the usual Fermi energy^74^). This corresponds to a metallic density that can be realized for example in experiments with aluminum^75^ at the electronic Fermi temperature of 12.53 eV. In the left panel of Figure 2, we show our new results for the static structure factor (SSF) *S*(*q*). More specifically, the dashed blue curve corresponds to the well-known random phase approximation (RPA), where the electronic density response to an external perturbation is treated on a mean field level.^58,74^ For the case of the UEG, the RPA becomes exact in the long-wavelength limit of *q* → 0, but it systematically underestimates the true SSF around the Fermi wavenumber *q* ∼ *q*_F_. These shortcomings have been corrected in the semiempirical effective static approximation (ESA),^72,73^ which is shown as the solid black line and is expected to give a highly accurate description of the UEG over the entire *q* range under these conditions. This is indeed verified by the green crosses depicting exact PIMC results for *S*(*q*) that have been obtained on the basis of simulations with *N* = 34 electrons without the ξ extrapolation method. Alas, these data are available only above a minimum wavenumber of *q*_min_ = 0.63*q*_F_, which is defined by the size of the simulation cell.^31^

**Figure 2:**
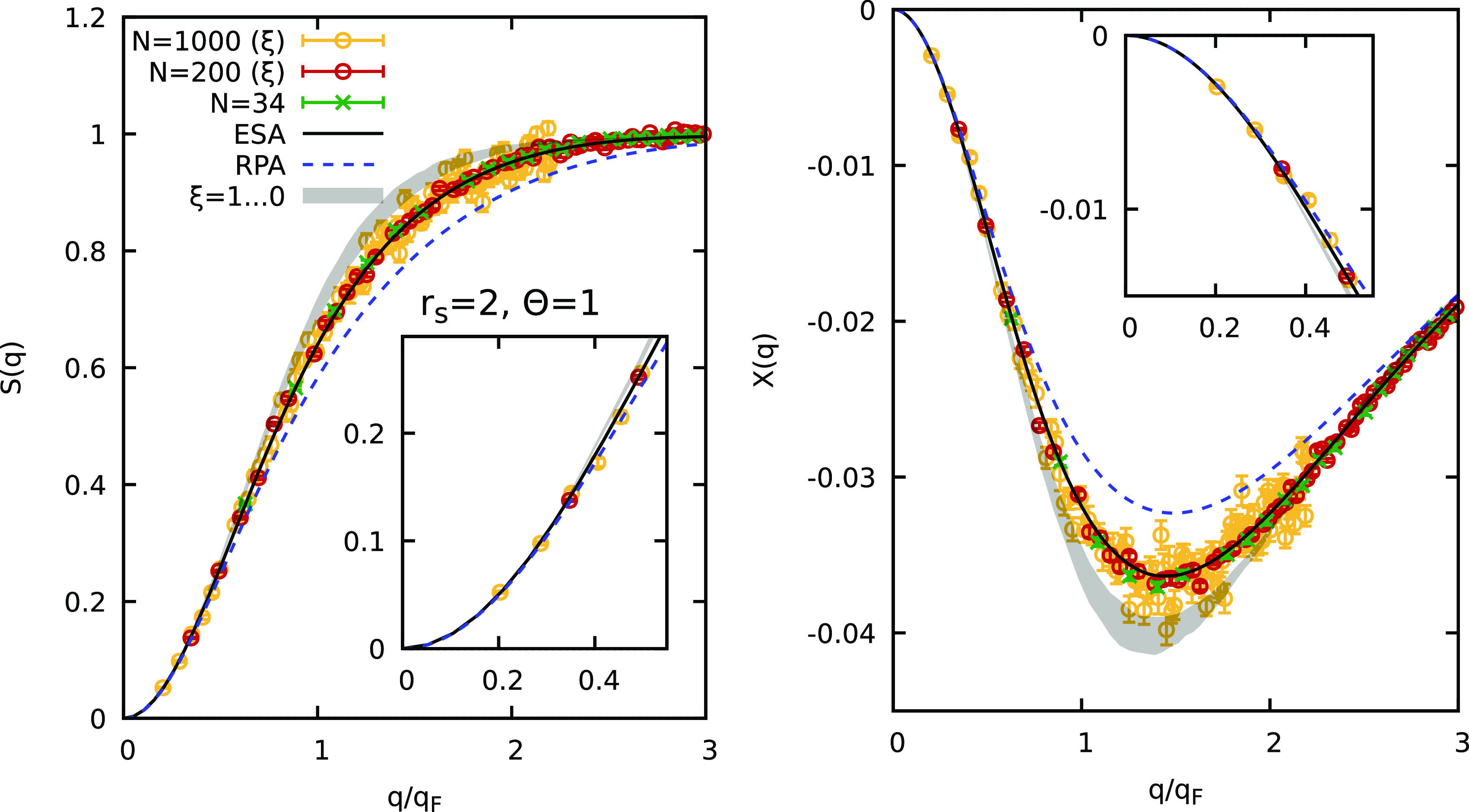
PIMC simulation of the warm dense UEG at *r*_s_ = 2 and Θ = 1. Static structure factor *S*(*q*) (left) and static linear density response function χ(*q*) (right) (see eq 3): dashed blue line, random phase approximation (RPA); solid black line, semiempirical effective static approximation (ESA);^72,73^ green crosses, exact PIMC results for *N* = 34; red circles (yellow stars), new ξ-extrapolated PIMC results for *N* = 200 (*N* = 1000). The insets show magnified segments around the long-wavelength limit.

To overcome this limitation, we have carried out extensive new calculations with *N* = 200 and *N* = 1000 electrons using the method explored in refs (61) and (62). In Figure 1, we show a snapshot from a corresponding PIMC calculation with *N* = 1000. A particular strength of the method is the full treatment of quantum diffraction effects over the entire length scale, as each electron is represented by an entire path of *P* = 200 positions along the imaginary-time domain; these paths would collapse to point particles in the classical limit of β → 0.

Coming back to the SSF shown in Figure 2, we find that the new results for *N* = 200 (red circles) and *N* = 1000 (yellow circles) that have been obtained on the basis of eq 2 using input data from the sign-problem free domain of ξ ≥ 0 (shaded gray area) are in excellent agreement with the ESA results and the exact PIMC results for *N* = 34, where they are available. In particular, we unambiguously demonstrate that this approach is capable of recovering the *q* → 0 limit, which is known exactly in the case of the UEG (see also the inset showing a magnified segment around this regime). The somewhat larger spread of the data points for the *N* = 200 and *N* = 1000 cases is due to the computational cost increasing with approximately *N*^2^ and thus the significantly reduced number of snapshots available for averaging with a manageable computational cost.

In the right panel of Figure 2, we repeat this analysis for static linear density response function χ(*q*), which can be computed from PIMC results for *F*(*q*, τ) based on the imaginary-time version of the well-known fluctuation–dissipation theorem^67,76^
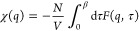
3

Overall, we find the same general trends as for *S*(*q*). The RPA underestimates the true magnitude of the density response around the Fermi wavenumber; the ESA is quasi-exact for all values of *q* and nicely agrees with the direct PIMC results, and most importantly, our new, large-scale PIMC simulations can predict the correct *q* → 0 limit (see also the inset).

To further highlight the high quality of these results, we consider the exact expression
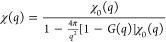
4where χ_0_(*q*) is the temperature-dependent Lindhard function describing the density response of an ideal Fermi gas^74^ and the complete, wavenumber-resolved, information about electronic exchange–correlation effects is included in static local field correction (LFC) *G*(*q*) .^77,78^ Therefore, the LFC constitutes key input for a number of applications, such as the computation of electrical conductivities,^79^ the interpretation of XRTS experiments,^80^ the development of thermal XC functionals,^81−84^ and the exchange–correlation kernel in time-dependent DFT simulations.^85,86^ In Figure 3, we show the LFC for the same conditions that were used in Figure 2. The solid blue line corresponds to the recent analytical parametrization of the ESA^73^ that includes the correct *q* → 0 limit given by the compressibility sum rule (CSR)
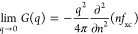
5where *f*_xc_ is the exchange–correlation energy of the UEG.^32,40,47,48^ In practice, we evaluate eq 5 using the parametrization by Groth et al.,^32^ and the results are included as the dashed–dotted yellow curve. Furthermore, the dashed black line shows the PIMC-based machine-learning (ML) representation of *G*(*q*; *r*_s_, Θ) from ref (77). The ESA and ML curves are very similar in the depicted *q* range and noticeably differ only in the single-particle limit of *q* ≫ *q*_F_. As usual, the green crosses show exact PIMC results for *N* = 34 that have been obtained by inverting eq 4, and the red circles show the corresponding ξ-extrapolated PIMC results for *N* = 200. Again, we find that the ξ extrapolation method very accurately describes this sophisticated exchange–correlation property over all length scales. This raises the intriguing possibility of studying long-range correlations in other Fermi systems such as warm dense hydrogen, where the true *q* → 0 limit is the subject of active investigations.^54,59,87^

**Figure 3:**
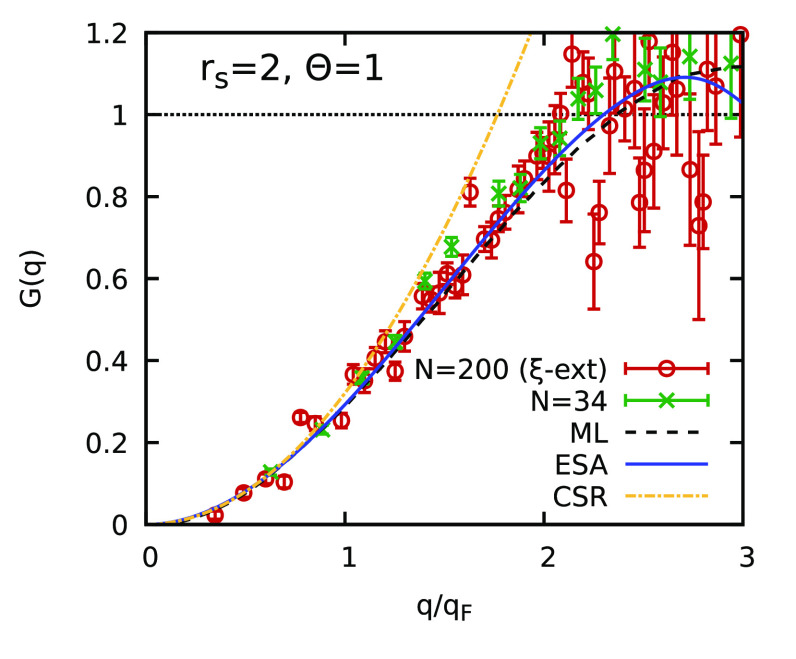
Local field correction *G*(*q*) (see eq 4) for *r*_s_ = 2 and Θ = 1: dashed black line, neural-net representation from ref (77); solid blue line, analytical parametrization of the ESA;^73^ dashed–dotted yellow line, compressibility sum rule (eq 5) evaluated from the parametrization by Groth et al.;^32^ green crosses, exact PIMC results for *N* = 34; red circles, new ξ-extrapolated PIMC results for *N* = 200.

As a second example, we consider the UEG at *r*_s_ = 10 and Θ = 0.5. These conditions correspond to the boundary of the strongly coupled electron liquid regime^63,64^ that is known to exhibit a wealth of interesting physical phenomena such as a nonmonotonic roton-type feature in the dynamic structure factor.^31,57,88−90^ In Figure 4, we show the corresponding SSF and static linear response function with the usual color code. To assess the accuracy of the ξ extrapolation under these conditions, we have carried out new, exact PIMC simulations for *N* = 14 electrons (green crosses). We find an average sign of *S* = 0.02, making them computationally involved, but still feasible.^23^ Considering the static structure factor, the PIMC reference results are in very good agreement with the ESA curve; small systematic deviations are visible only in the vicinity of the peak, where the latter slightly overestimates the true SSF. The red circles show the ξ-extrapolated results, which nicely agree with the green crosses everywhere. Interestingly, we find that the effect of quantum statistics on *S*(*q*) is small (see the shaded gray area indicating the sign-problem free domain of ξ ≥ 0), even though the degree of cancellations of positive and negative terms is substantial already for the relatively small system size of *N* = 14. A similar observation has been reported recently for the case of ultracold ^3^He,^11^ which, too, constitutes a strongly coupled quantum liquid. Finally, the yellow circles show new, ξ-extrapolated results for *N* = 200 electrons, and we find the same good performance as for the case of *r*_s_ = 2 investigated above.

**Figure 4:**
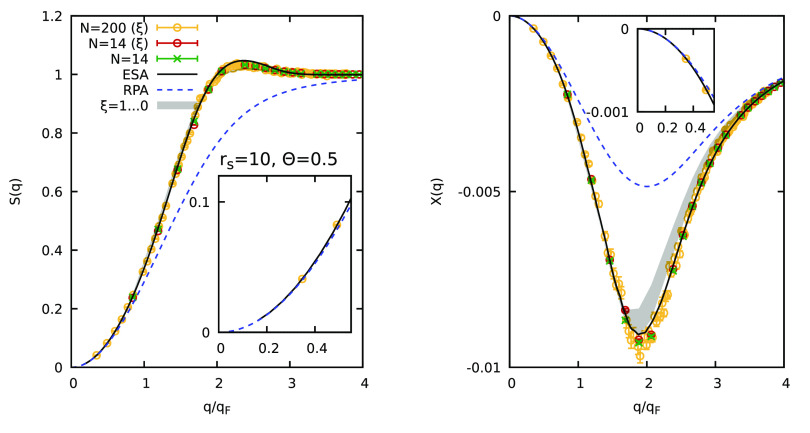
PIMC simulation of the warm dense UEG at *r*_s_ = 10 and Θ = 0.5. Static structure factor *S*(*q*) (left) and static linear density response function χ(*q*) (right) (see eq 3): dashed blue line, random phase approximation (RPA); solid black line, semiempirical effective static approximation (ESA);^72,73^ green crosses, exact PIMC results for *N* = 14; red and yellow circles, ξ-extrapolated PIMC results for *N* = 14 and *N* = 200, respectively. The insets show magnified segments around the long-wavelength limit.

The right panel of Figure 4 shows the results for static linear density response function χ(*q*). First, we observe that the impact of quantum statistics is considerably larger than that for *S*(*q*) = *F*(*q*, 0). Indeed, eq 3 implies that χ(*q*) directly depends on the imaginary-time diffusion of the electrons, which is more sensitive to quantum effects than the static structure of the system. Second, we find that the ξ extrapolation based on eq 2 becomes somewhat inaccurate when the full sign-problem free interval of ξ ∈ [0, 1] is used. This is investigated in more detail in Figure 5, where we show the ξ dependence of χ(*q*) at *q* ≈ 2*q*_F_. Specifically, the blue diamonds show exact PIMC results, which are available as a benchmark over the full ξ range in this case, and the various curves correspond to quadratic extrapolations based on eq 2 using as input data within different the interval ξ ∈ [0, ξ_max_]. The dashed black line corresponds to the usual choice of ξ_max_ = 1 and underestimates the true density response in the Fermionic limit of ξ = −1 by ∼1%. We note that this is still a reasonable level of accuracy for the description of a strongly coupled Fermi liquid but is considerably worse compared to the WDM case investigated above. The dashed–dotted yellow and dotted green curves correspond to ξ_max_ = 0.7 and ξ_max_ = 0.5, respectively, and exhibit a substantially improved agreement with the PIMC data for ξ < 0. Empirically, these values of ξ seem to be equally reasonable, and we have used ξ = 0.5 to compute the red circles in the right panel of Figure 4. Evidently, this truncated extrapolation scheme works very well over the entire *q* range. Finally, the solid red curve in Figure 5 corresponds to ξ_max_ = 0.3. In this case, the ξ extrapolation is based on only four data points, which leads to a larger uncertainty compared with the other analyzed fitting intervals.

**Figure 5:**
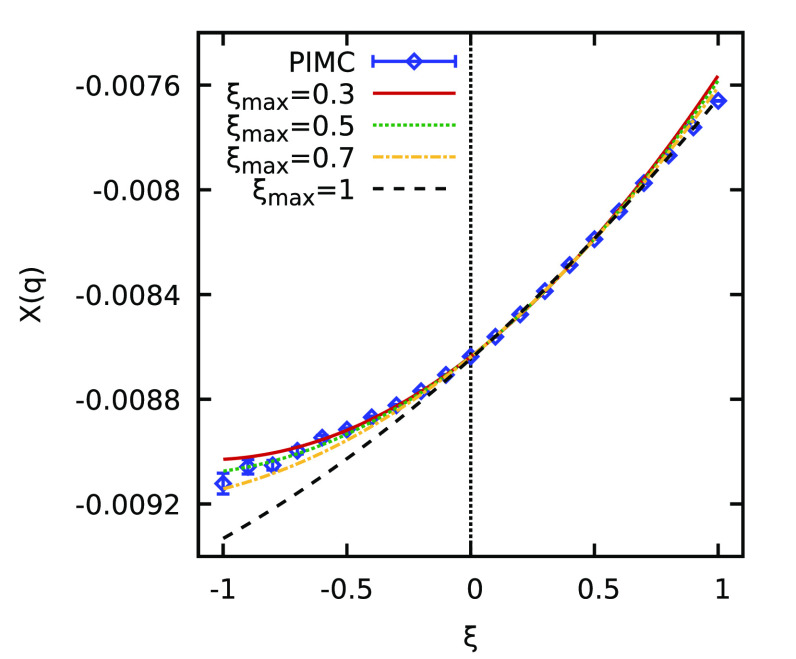
ξ dependence of static linear density response function χ(*q*) for *N* = 14, *r*_s_ = 10, and Θ = 0.5 at *q* ≈ 2*q*_F_. The blue diamonds depict the exact PIMC results, and the various curves have been obtained by quadratic fits based on eq 2 using as input data in the interval of ξ ∈ [0, ξ_max_].

Returning to the static linear density response function shown in Figure 4, we find that the effect of quantum statistics is restricted to the range 1.75*q*_F_ ≲ *q* ≲ 4*q*_F_ for these conditions. Unsurprisingly, the ξ-extrapolated results for *N* = 200 electrons (yellow circles) are in excellent agreement with the solid black ESA curve everywhere and reproduce the correct *q* → 0 limit (see also the inset). We thus conclude that the ξ extrapolation method is applicable beyond the WDM regime and constitutes a valuable tool for the investigation of strongly coupled Fermi liquids.

In this work, we have presented extensive new *ab initio* PIMC simulations of the UEG with an unprecedented system size (*N* ≤ 1000 electrons). In this way, we have unambiguously demonstrated that the ξ extrapolation method^61,62^ can describe non-ideal Fermi systems over all length scales, including the long-range limit (*q* → 0) that is known exactly in the case of the UEG, but not for other systems such as warm dense two-component plasmas or ultracold atoms. From a physical perspective, we have considered both the WDM regime that is of high current interest, e.g., for the description of laser fusion applications and astrophysical models, and the strongly coupled electron liquid regime, which exhibits phenomena such as the roton-type feature of the dynamic structure factor that is interesting in itself. We are convinced that these findings open up a host of new avenues for impactful future research in a wide variety of research fields.

First, an intriguing application of the ξ extrapolation method stems from its unique capability to accurately probe even the largest length scales of the UEG. Naturally, when the long-wavelength limit is approached, otherwise inaccessible information is unlocked that concerns specific thermodynamic quantities as well as transport coefficients. (a) The *q* → 0 limit of the static local field correction directly leads to the isothermal compressibility via the eponymous sum rule without the need for thermodynamic integration or differentiations.^74^ Such a thermodynamic route that is independent of the internal energy would provide a rigorous check for existing UEG equations of state^32,48^ that are typically free energy representations whose derived thermodynamic properties are known to suffer from certain pathologies.^52^ (b) The frequency-dependent electrical conductivity is connected to the long-wavelength limit of the current response function, while the thermal conductivity is connected to the hydrodynamic limit of the heat (or energy) current response function (see the respective Kubo and Green–Kubo formulas^91,92^). (c) Within the current density version of linear response theory, it is known that the generalized viscoelastic coefficients are connected to long-wavelength limits that involve the respective longitudinal and transverse dynamic local field corrections.^93,94^ Moreover, the shear viscosity is connected to a hydrodynamic limit that involves the transverse local field correction.^74,93,94^ (d) Within the spin-resolved density version of linear response theory, it is known that the static spin susceptibility is connected to the *q* → 0 limit of the static spin-antisymmetric local field correction.^95^ In addition, the spin diffusion coefficient/constant is connected to a hydrodynamic limit that involves the dynamic spin-antisymmetric local field correction.^91^

More importantly, the ξ extrapolation method will allow for the first time the study of long-range phenomena in real WDM systems based on *ab initio* PIMC simulations starting with hydrogen.^96−100^ This will facilitate the interpretation of XRTS experiments in a forward scattering geometry, where the system is effectively probed on large length scales. From a physical perspective, one can expect that such measurements will be highly sensitive to the density of the probed system and, in this way, will complement backward scattering measurements that are particularly sensitive to parameters such as the temperature and ionization.^101−103^ In accordance with this reasoning, a second potential application of the ξ extrapolation method that is related to the study of WDM concerns the estimation of thermodynamic quantities and transport coefficients such as thermal conductivity^60^ and electrical conductivity,^59^ which may substantially depend on exchange–correlation effects.^54^

Returning to the UEG itself, we find it might be interesting to study the spin-resolved density response, as well as the spin-resolved components of the static structure factor that, in contrast to the spin-averaged χ(*q*) and *S*(*q*), are not described properly by the RPA or by more sophisticated dielectric schemes^104−110^ even in the long-range limit of *q* → 0.^111^ Such a study might provide new insights into the performance of different approximations to the LFC, and it can provide useful input and benchmark data for other applications.

A further interesting line of research is the application of the ξ extrapolation method to other systems. On the basis of our encouraging results for the strongly coupled electron liquid, we propose to utilize the method for ultracold atoms such as the short-range interacting ^3^He, but also dipolar systems.^23,112^ For these systems, interesting long-range phenomena include the acoustic mode in the dynamic structure factor for small *q* values and the long-wavelength limit of the SSF that is given by the compressibility sum rule.^113^ Furthermore, we note that the method has already been applied to electrons in quantum dots in previous studies.^61,62,114^ Indeed, it is well-known that trapped Fermi systems exhibit interesting effects such as the formation of Wigner molecules^115,116^ and a negative superfluid fraction.^16,112,117^

Finally, we stress that, despite its impressive performance, the ξ extrapolation method is a very new technique, and its further methodological improvement remains in its infancy compared to more established methods such as restricted PIMC.^25,118,119^ The observed improvement of the extrapolation due to the truncation of the ξ interval in the sign-problem free domain for *r*_s_ = 10 and Θ = 0.5 suggests that much can be gained by developing better extrapolation schemes. In addition, the absence of the exponential bottleneck poses new challenges, which might be met by combining PIMC with other ideas such as the adaptive long-range potential scheme that has been suggested recently in ref (120) or the quantum ring-polymer contraction method that has been introduced in the context of PIMD.^121^

## Supporting Information Available

Transparent Peer Review report available (PDF)
