# Supplementary material for: Ab Initio Path Integral Monte Carlo Simulations of the Uniform Electron Gas on Large Length Scales
Source: J Phys Chem Lett. 2024 Jan 29;15(5):1305–13. doi: 10.1021/acs.jpclett.3c03193 (PMC10860150; doi:10.1021/acs.jpclett.3c03193)
Supplement: Supplementary file 1 — jz3c03193_si_001.pdf [file jz3c03193_si_001.pdf]

jz-2023-03193k.R1

Name: Peer Review Information for "<i>Ab Initio</i> Path Integral Monte Carlo Simulations of the Uniform Electron Gas on Large Length Scales"

First Round of Reviewer Comments

Reviewer: 1

Comments to the Author

Article jz-2023-03193k

Report

Ab initio path integral Monte Carlo simulations of the uniform electron gas on large length scales

By Tobias Dornheim, Sebastian Schwalbe, Zhandos A. Moldabekov, Jan Vorberger and Panagiotis Tolias

This paper is interesting with new results and I would like to see it publish in the Journal of Physical Chemistry Letters. However there are a number of concerns and possibility for improvement, which have to be done before publication.

In the present work to overcome the sign problem, the authors use technique allowing to get information about the fermionic system as extrapolation of results obtained within the bosonic system The authors use this technique to carry out very large path integral Monte Carlo simulations of the warm dense electron gas and demonstrate that it is capable of providing a description of the thermodynamic functions such as static structure factor, the static density response function, and local field correction.

The main contributions of this paper are data from numerical simulations however the authors need to provide specific details of the article's scientific background so that their scientific significance can be properly judged and appreciated. There are a number of concerns and possibility for improvement.

Some specific instances are:

1)To do extrapolation of the thermodynamic functions from bosons to fermions the authors introduced in the quantum symmetrized partition function the new formal continuous variable $\xi \in [-1, 1]$. As result the partition function depends on the new parameter ξ^Npp, where Npp is number of transposition in expansion of each permutations. The authors claimed: In addition, the factor ξ^Npp takes into account the impact of quantum statistics, with $\xi = 1$ corresponding to bosons, $\xi = -1$ to fermions, and $\xi = 0$ to hypothetical distinguishable quantum particles that are often being referred to as boltzmannons in the literature ...AND... the new partition function is a continuous function of combination ξ^Npp.

But for ξ=0 the single nonzero contribution to partition functions comes from the identical permutation with Npp=0. So the partition function contains the factor 0^0. The expression 0^0 (zero to the power of zero) is considered by many textbooks to be vague and meaningless [1-4]. This is related to the topic, what is the function f(x,y)=x^y of the two variables at point (0, 0) as it has an irremovable discontinuity. In fact, along the positive X-axis direction, where y=0, it is equal to one, and along the positive direction of the Y axis, where x = 0, it is equal to zero. Therefore, no agreement can produce a continuous function at (0, 0) for extrapolation throughout $\xi \in [-1, 1]$. Definition debate on the 0^0 has been going on since at least the early 19th century (see for example [1-4]). This problem has to be mentioned and discussed in this article.1.Bourbaki. Theory of Sets // Elements of Mathematics, Springer-Verlag, 2004, III, § 3.5. 2.Augustin-Louis Cauchy. Cours d'Analyse de l'Ecole Royale Polytechnique (1821). In his Oeuvres Completes, series 2, volume 3. 3.Guillaume Libri. Note sur les valeurs de la fonction 0^0, Journal fur die reine und angewandte Mathematik 6 (1830), 67-72. 4.Guillaume Libri. Memoire sur les fonctions discontinues, Journal fur die reine und angewandte Mathematik 10 (1833), 303—316.

2)The literature review has to be extended by the several possibilities to overcome the sign problem.

2a)In articles [1,2] to overcome the ` ` sign problem'' the exchange interaction in the density matrix is expressed through a positive semidefinite Gram determinant of the Fermi system, which allows to avoid the sampling of a bosonic partition function for obtaining fermionic expectation values. Due to the Gram determinant, this approach does not suffer from the 'fermionic sign problem'. 1) V. Filinov, P. Levashov and A. Larkin, J. Phys. A Math. Theor. 55 (3), 035001 (2022). 2)V. S. Filinov, R. S. Syrovatka & P. R. Levashov (2022) Solution of the 'sign problem' in the path integral Monte Carlo simulations of strongly correlated Fermi systems: thermodynamic properties of helium-3, Molecular Physics, 120:14, e2102549 DOI: 10.1080/00268976.2022.2102549

2b) An alternative approach based on the Wigner formulation of quantum mechanics in the phase space was used in [3,4] to avoid the antisymmetrization of matrix elements and hence the ` ` sign problem''. This approach allows to reproduce the Pauli blocking of fermions by the effective pair pseudopotential in phase space and it is able to calculate quantum momentum distribution functions, transport properties and average values of quantum operators in the phase space. 3.A.Larkin, V. Filinov, and V. Fortov, Journal of Physics A: Mathematical and Theoretical 51, 035002 (2017) 4. W. Ebeling, V.Fortov, and V. Filinov, Quantum Statistics of Dense Gases and Nonideal Plasmas (Springer, Berlin, 2017).

2c) As it follows from [5] the exchange pseudopotential in coordinate space results in the fermi repulsion of fermions and the bose attraction of bosons. These effective interactions are the effects of quantum statistics. So the analytical behaviors of thermodynamic functions has to be different for fermion like particles ($\xi<0$) and for boson like ones ($\xi>0$) and extrapolation throughout the fermionic sector $\xi \in [-1, 0]$ of the results obtained within the bosonic system at $\xi \in [0, 1]$ is problematic for degenerated systems. This problem has to be mentioned and discussed in this article. 5) Huang, K. Statistical Mechanics; John Wiley & Sons: Hoboken, NJ, USA, 1963.

Author's Response to Peer Review Comments:

Dear Editor,

please find our detailed response in the file "Response.pdf".

Sincerely,

Tobias Dornheim

Response to referee report
“*Ab initio* Path Integral Monte Carlo Simulations of the Uniform Electron Gas on Large Length Scales”

Tobias Dornheim Sebastian Schwalbe Zhandos Moldabekov
Jan Vorberger Panagiotis Tolias

December 13, 2023

Dear Editor,

we would like to thank the referee for reviewing our work, and for the positive and constructive feedback. Please find our in-depth response to the individual points, as well as our response to the non-scientific Editorial requests below. For convenience, we also include a diff file highlighting all changes.

We hope that the revised ms is now suitable for publication in the Journal of Physical Chemistry Letters.

Sincerely,

T. Dornheim, for the authors

Referee 1

> This paper is interesting with new results and I would like to see it publish in the Journal of Physical Chemistry Letters. However there are a number of concerns and possibility for improvement, which have to be done before publication.
>
> In the present work to overcome the sign problem, the authors use technique allowing to get information about the fermionic system as extrapolation of results obtained within the bosonic system The authors use this technique to carry out very large path integral Monte Carlo simulations of the warm dense electron gas and demonstrate that it is capable of providing a description of the thermodynamic functions such as static structure factor, the static density response function, and local field correction.
>
> The main contributions of this paper are data from numerical simulations however the authors need to provide specific details of the article’s scientific background so that their scientific significance can be properly judged and appreciated. There are a number of concerns and possibility for improvement.

We thank the referee for reviewing our manuscript, and the overall very positive and encouraging assessment.

> 1)To do extrapolation of the thermodynamic functions from bosons to fermions the authors introduced in the quantum symmetrized partition function the new formal continuous variable $\xi \in [-1, 1]$. As result the partition function depends on the new parameter $\xi^{N_{pp}}$, where N_{pp} is number of transposition in expansion of each permutations. The authors claimed: In addition, the factor $\xi^{N_{pp}}$ takes into account the impact of quantum statistics, with $\xi = 1$ corresponding to bosons, $\xi = -1$ to fermions, and $\xi = 0$ to hypothetical distinguishable quantum particles that are often being referred to as boltzmannons in the literature ...AND... the new partition function is a continuous function of combination $\xi^{N_{pp}}$.
>
> But for $\xi = 0$ the single nonzero contribution to partition functions comes from the identical permutation with $N_{pp} = 0$. So the partition function contains the factor 0^0. The expression 0^0 (zero to the power of zero) is considered by many textbooks to be vague and meaningless [1-4]. This is related to the topic, what is the function $f(x, y) = x^y$ of the two variables at point $(0, 0)$ as it has an irremovable discontinuity. In fact, along the positive X-axis direction, where $y = 0$, it is equal to one, and along the positive direction of the Y axis, where $x = 0$, it is equal to zero. Therefore, no agreement can produce a continuous function at $(0, 0)$ for extrapolation throughout $\xi \in [-1, 1]$. Definition debate on the 0^0 has been going on since at least the early 19th century (see for example [1-4]). This problem has to be mentioned and discussed in this article.1.Bourbaki. Theory of Sets // Elements of Mathematics, Springer-Verlag, 2004, III, § 3.5. 2.Augustin-Louis Cauchy. Cours d'Analyse de l'Ecole Royale Polytechnique (1821). In his Oeuvres Completes, series 2, volume 3. 3.Guillaume Libri. Note sur les valeurs de la fonction 0^0, Journal fur die reine und angewandte Mathematik 6 (1830), 67-72. 4.Guillaume Libri. Memoire sur les fonctions discontinues, Journal fur die reine und angewandte Mathematik 10 (1833), 303|316.

We agree with the referee that the particular value for 0^0 is not a-priori clear. In our context, we set $\xi^0 \equiv 1$ as a matter of definition for all values of ξ. A corresponding explanation has been added under Eq. (1).

> 2)The literature review has to be extended by the several possibilities to overcome the sign problem.
>
> 2a)In articles [1,2] to overcome the ‘‘sign problem’’ the exchange interaction in the density matrix is expressed through a positive semidefinite Gram determinant of the Fermi system, which allows to avoid the sampling of a bosonic partition function for obtaining fermionic expectation values. Due to the Gram determinant, this approach does not suffer from the ‘fermionic sign problem’. 1) V. Filinov, P. Levashov and A. Larkin, J. Phys. A Math. Theor. 55 (3), 035001 (2022). 2)V. S. Filinov, R. S. Syrovatka and P. R. Levashov (2022) Solution of the ‘sign problem’ in the path integral Monte Carlo simulations of strongly correlated Fermi systems: thermodynamic properties of helium-3, Molecular Physics, 120:14, e2102549 DOI: 10.1080/00268976.2022.2102549
>
> 2b) An alternative approach based on the Wigner formulation of quantum mechanics in the phase space was used in [3,4] to avoid the antisymmetrization of matrix elements and hence the ‘‘sign problem’’. This approach allows to reproduce the Pauli blocking of fermions by the effective pair pseudopotential in phase space and it is able to calculate quantum momentum distribution functions, transport properties and average values of quantum operators in the phase space. 3.A.Larkin, V. Filinov, and V. Fortov, Journal of Physics A: Mathematical and Theoretical 51, 035002 (2017) 4. W. Ebeling, V.Fortov, and V. Filinov, Quantum Statistics of Dense Gases and Nonideal Plasmas (Springer, Berlin, 2017).

We thank the referee for pointing out these relevant references and now appropriately cite them in the introduction.

> 2c) As it follows from [5] the exchange pseudopotential in coordinate space results in the fermi repulsion of fermions and the bose attraction of bosons. These effective interactions are the effects of quantum statistics. So the analytical behaviors of thermodynamic functions has to be different for fermion like particles ($\xi < 0$) and for boson like ones ($\xi > 0$) and extrapolation throughout the fermionic sector $\xi \in [-1, 0]$ of the results obtained within the bosonic system at $\xi \in [0, 1]$ is problematic for degenerated systems. This problem has to be mentioned and discussed in this article. 5) Huang, K. Statistical Mechanics; John Wiley and Sons: Hoboken, NJ, USA, 1963.

We agree with the referee and have added a corresponding explanation below Eq. (2) of the main text.

Editorial Requests

> 1) Graphics: One or more of your figures and tables includes a reference citation. Please confirm that this pertains only to data and not the figure itself. If it pertains to the use of a published image, permissions must be secured for any graphics NOT originally published by ACS or for Open Access content which permits reuse with credit only. Permission is needed if you are using another publisher's or copyright owner's figures/tables verbatim, adapting/modifying them, or using them in part. If the images are from an Open Access publisher that does not require permission for reuse, please confirm.

Reference citations only pertain to data; no additional permissions are required.

> 2) Title: In the main manuscript file, set the title in title case, with the first letter of each principal word capitalized.

Done.

> 3) TOC Graphic: Please resize the TOC graphic per journal guidelines (2 in x 2 in).

Done.

> 4) References: In both the main file and the supporting information, fix the style of all references to use JPCL formatting (check all references carefully). ***JPC Letters reference formatting requires that journal references should contain: () around numbers; author names; article title (titles entirely in title case or entirely in lower case); abbreviated journal title (italicized); year (bolded); volume (italicized); and pages (first-last). Book references should contain author names; book title (in the same pattern); publisher; city; and year. Websites must include date of access.

We have checked all references and have amended the information as requested. Please note that we use LaTeX+BibTeX with the given ACS template.

jz-2023-03193k.R2

Name: Peer Review Information for "<i>Ab Initio</i> Path Integral Monte Carlo Simulations of the Uniform Electron Gas on Large Length Scales"

Second Round of Reviewer Comments

Reviewer: 1

Comments to the Author

I am not satisfied by the two lines formal answers on my comments. I'd like to read related detailed discussions included in article.

Author's Response to Peer Review Comments:

Dear Editor, dear Referee,

we regret to inform you that we cannot accommodate the referee's unreasonable demands. During the review process, the referee focused on two main points:

1) The potentially undefined nature of the term "0^0".

In response to the referee, we clearly state that we define this term as 0^0=1 in the ms, and explain the physical reason for this choice. Moreover, we followed the referee's advice and give a reference to a textbook, where the issue of "0^0" is discussed from a mathematical perspective. Any further discussion of this exclusively mathematical question is clearly beyond the scope of a physical chemistry letter.

2) Extending the literature review.

The referee suggested "including" a number of past works on fermionic quantum Monte Carlo (QMC) simulations mostly by the same group of authors: V. Filinov, A. Larkin, and P. Levashov. In response to the referee, we added these references to the introduction. We stress that, this being a letter format, no single QMC method is explicitly discussed by us here, including highly important (and well cited) concepts such as the fixed-node approximation. The referee's current demand "I'd

like to read related detailed discussions included in article.”, is thus highly inappropriate. In general, we would like to note that pushing one’s own work---or that of a close colleague---during peer review is questionable.

To summarize, we feel that we have sufficiently replied to and resolved all the points raised by the referee.

Sincerely,

T. Dornheim, for the authors
